# Heterogeneous global health stock and growth: quantitative evidence from 140 countries, 1990–2100

**DOI:** 10.1186/s13690-018-0327-8

**Published:** 2018-12-28

**Authors:** Isma Addi Jumbri, Shinya Ikeda, Shunsuke Managi

**Affiliations:** 1 Graduate School of Environmental Studies, Tohoku University, Sendai, Japan; 2 College of Agriculture, Regional and Environmental Science, Ibaraki University, Inashiki, Japan; 3 Department of Urban and Environmental Engineering, and Urban Institute, Kyushu University, Fukuoka, Japan

**Keywords:** Sustainable development, Health stock, Health forecasting, Time series

## Abstract

**Background:**

In the prevailing economic perspective, health is viewed as a type of capital stock that yields ‘healthy days’ in human society. However, evaluations of this health capital stock are still limited to specific contexts. The primary aim of this study is to measure and forecast the global health stocks in 140 countries from 1990 to 2100.

**Methods:**

The health capital stock in each country from 1990 to 2015 was estimated using a capital approach. The future health stocks between 2016 and 2100 were forecast using a time-series model.

**Results:**

Based on the health stocks from 1990 to 2015, low-income countries have much larger and more rapidly growing health stocks. In the long-term, to 2100, upper-middle income countries, particularly countries in the Middle East and North Africa, exhibit great growth that benefits from the peaks in their youth or working-age populations. Immigration also contributes to health stock growth, as do other factors, e.g., the fertility rate, population ageing, and working-age and youth populations.

**Conclusions:**

Health stock is a vital component of global sustainable development that should be consistently included as a stock-based sustainability index in the evaluations of other capital to accurately measure national wealth and sustainability.

## Background

Human capital, which consists of human health and education, is an important factor for economic growth as well as other factors, such as income, labour productivity, saving and investment, and demographic structure [1–4]. Although human health and education are important factors for us, health status has larger positive effects on the economic growth in Sub-Saharan Africa relative to education status [5]. Following the importance of national health status, the improvement could benefit not only human capital, but also contributes toward economic sustainability throughout the world. In global dispute, Sustainability Development Goals (SDGs) of the 2030 Agenda for Sustainable Development focuses specifically on ensuring healthy lives and promoting the well-being of individuals of all ages. Then, the SDGs cannot be achieved unless the prevalence of debilitating illnesses is low, and a population’s health can be maintained with ecologically sustainable development [6–9]. According to the concern, sustainability based on health status has become a central criterion used by all parties, including foundations and governmental or international agencies as well as evaluations of public health programmes and global health [10]. The World Health Organization (WHO) [11] also argued that health is positioned as a major contributor to the SDGs as follows: without health, many other SDGs cannot be achieved; simultaneously, health also benefits from achieving progress towards the other SDGs.

Since achieving the health-related SDGs require the criteria of sustainability, measurement of health status is of particular interest by scholars and policy makers. They would use various indicators of health status, e.g., fertility rate, disability-adjusted life year (DALY), and healthy life expectancy. However, the indicators are just reflected by a few aspects of health status, and not enough to consider the availability of substitute and complementary resources for improving health status. The latter deficit is critical for achieving SDGs due to its broad targets under limited global resources. Those problems could be solved by using a simple indicator, Inclusive Wealth Index (IWI) [12].

The Inclusive Wealth Report 2012 (IWR 2012) quantified health status as a vital form of wealth by estimating the value of the improvement in life expectancy over a nineteen-year period from 1990 to 2008. The report demonstrated that certain countries are advancing the three pillars of sustainability, i.e., social, environmental and economic. For the practical measurement of IWI, Arrow et al. [13] proposed a measure of health capital that is consistent with theories of public economics; for example, the amount of health stock can be measured by the total discounted years of life expectancy in a country’s population. This measure can suggest the substitution between the health capital and other types of capital, such as natural capital and produced capital.

However, health capital not only must be measured as shown in IWR 2012 but also needs to be forecast, particularly to achieve SDG 3 of the SDGs, which focuses on healthy lives and well-being. Concerns about health care expenditure growth and its long-term sustainability have also risen to the top of policy agendas in many countries that aim to launch forecasting projects to support policy planning [14]. Along the necessity of forecasting the population health, the related studies have advanced over time and increased in sophistication in many specialized areas, including economics, technology, politics, environmental fields, and the field of public health [15].

To fill the gap between previous research and the above requirement, in this study, we aim to measure and forecast the national health capital stock in global perspective. First, we measure the health-stock index, as proposed by Arrow et al. [13], in 140 countries from 1990 to 2015. Our measurement covers more countries and time-span than in IWR 2012. Then, based on our previously measured historical health-stock data, we forecast the future values using a technique of autoregressive integrated moving average (ARIMA). So, this approach has been extensively used for health forecasting [14–17] because the ARIMA model is a prediction method with a sophisticated statistical theory and the strong adaptive ability [18]. We can analyse growth patterns of the health stock in 140 countries in the future of the twenty-first century, as well as the linkage between sustainable development and improvement in health and population growth, particularly in low-income countries (LICs) where many people still face severe health conditions. For instance, people in LICs are often prevented to access healthcare (medicine and devices) and require for the improvement of the quality of health [19, 20]. Thus, this research contributes toward building up the criteria for health-related sustainability in LICs and the other countries.

The remainder of the paper is organized as follows. Next section describes the methodology applied to model and forecast the health stock. Section 3 depicts the health-stock results from 140 countries from 1990 to 2015 and the forecasting trends from 2016 to 2100. Section 4 we discuss implications for achieving sustainable society from the heterogeneous health stock condition. The final section provides summaries and discusses the implications of this study with regard to how the SDGs can be achieved.

## Methods

We briefly explain our method of global health-stock estimation since we basically apply the capital approach proposed by Arrow et al. [13]. The capital approach evaluates human wealth as total current values of human, produced, and natural capital. This approach has been applied in national wealth evaluation [12, 21], regional health evaluation [22], and furthermore in project evaluation [23, 24]. Using this method, the amount of health stock can be calculated by the total discounted years of life expectancy for each age group in a country’s population. Note that for monetarizing the health stock we can use the value of an additional year of life, the VSL, although it doesn’t affect the change rates of health capital due to its constant assumption.

Let π(*a*) be the proportion of people of age a and *f*(*T*| *T* ≥ *a*) be the conditional probability density of death at age T given survival to age *a*. The conditional probability density results from computing the probability density that someone born will die at age *T*, *f* (*T*) and the corresponding cumulative distribution at age *a*, *F*(*a*) as follows:

$$ f\left(T|T\ge a\right)=\frac{f(t)}{1-F(a)} $$

We assume that *δ* is the discount rate of 0.05 for future survival years, and the value of an additional year is independent of age. Then, the amount of health stock per capita at age *a*, *H*(*a*), is estimated as follows:

$$ H(a)={\sum}_{a=0}^{100}\pi (a)\left\{{\sum}_{T=a}^{100}f\left(T|T\ge a\right)\left({\sum}_{t=0}^{T-a}{\left(1-\delta \right)}^t\right)\right\} $$

Subsequently, the total amount of health stock can be obtained by summing it as $\sum \limits_{a=0}^{100}H(a)$ in the total population of a country.

The data of the probability of death at age t, *f*(*t*) by five-year age intervals, are obtained from life tables based on each country’s mortality and global health estimates, particularly the data regarding the number of people dying between ages x and x + n in each complete year from 2000 to 2015 by the WHO. Moreover, the WHO provides estimated life tables for the years 1990, 1995, 2000, 2005, 2010 and 2015. We used linear interpolation to obtain the missing values from 1991 to 1994,1996–1999, 2001–2004, 2006–2009, and 2011–2014. These data are available on the WHO website. The population data for each age and country were obtained from the UN. We used the data of the total population (both sexes combined) by five-year age groups for each country for the years 1990–2015. The data sources used to calculate the health stock are summarized in Table 1.

**Table 1:** Data sources used to calculate the health stock

No	Data	Explanation	Sources
1.	Population by age	Data provided by the United Census Bureau. We used population data by five-year age groups and both sexes for each country (140 countries) for the year 1990–2015.	https://www.census.gov/data-tools/demo/idb/region.php?N=%20Results%20&T=10&A=separate&RT=0&Y=2018&R=1&C=US
2.	Probability of dying by age	The probability of dying by age also refers to the mortality age. We used the number of people dying between ages x and x + n (ndx) for the years 1990–2015.	http://apps.who.int/gho/data/node.imr.LIFE_0000000032?lang=en

Next, we estimate the future health stock by using an econometric method. To forecast health stock, we applied ARIMA because it is commonly and widely used in a time series analysis [16, 25–27]. The ARIMA model also has the ability to use non-stationary time-series data, and many researchers use this model to forecast various health and medical phenomena [28]. For instance, the ARIMA had been used to forecast future monthly incidence of malaria (2018–2019) in the Kumasi Metropolis [29].

The ARIMA model represents a popular and flexible class of forecasting and represents a specific subset of univariate modelling in which a time series is expressed as a linear combination of prior values and/or lags in forecast errors [30]. Additionally, this model does not involve independent variables and uses information from the series to generate the forecast because the ARIMA model depends on the autocorrelation pattern in the series [31].

ARIMA econometric modelling considers historical data and decomposes the data into an autoregressive (AR) process including a memory of prior events and an integrated (I) process that stabilizes or renders the data stationary, enabling easier forecasting and calculation of the moving average (MA) of forecast errors such that the longer the historical data, the more accurate the forecast because of learning over time [32]. The general form ARIMA model may possibly include autoregressive (p) terms, differencing (d) terms and moving average (q) operation and is represented by ARIMA (p, d, q). This study, ARIMA (p, d, q), which is a non-seasonal ARIMA, is used, and the mathematical formula for the ARIMA model can be expressed as follows:

$$ {Y}_t=c+{\varnothing}_1{Y}_{t-1}+\dots +{\varnothing}_p{Y}_{t-p}+{\theta}_1{e}_{t-1}+\dots +{\theta}_q{e}_{t-q}+{e}_t $$

Where

- *Y*_*t*_ = variable explained in time t;
- c = constant or intercept;
- ∅ = coefficient of each parameter p;
- θ = coefficient of each parameter q; and
- *e*_*t*_ = residuals or errors in time t.

The ARIMA models were analysed using the Box-Jenkins approach. In general, there are four stages in estimating an ARIMA model.

1. The identification of the model involves selecting the best fitting value of the *p, d* and *q* model, which refers to the number of AR lags, MA lags and differences, respectively. The Auto-Correlation Function (ACF) and Partial Auto-Correlation Function (PACF) are used to identify the best model. In our case, there are 140 countries, and we must identify the best model for each country. The best model for each country is summarized by the set of p, d, and q in Table 2.
2. After identifying the model, the estimation stage begins. We estimate the parameters of the ARIMA model.
3. The third stage is the diagnostic-checking. During this stage, a test for autocorrelation is performed. This procedure determines the statistical suitability of the model chosen in the previous steps. If this procedure fails, the process returns to the previous steps. Models that fail in these procedures should be rejected.
4. Using the estimated ARIMA parameters, we forecast for future periods. In this study, we forecast for the years 2016 to 2100 using data previously estimated from 1990 to 2015.

**Table 2:** ARIMA model (p, d, q) for each country

ARIMA model (p,d,q)	Country	No. of Country
(2, 1, 0)	Afghanistan, Armenia, Estonia, Finland, Gabon, Haiti, Jamaica, Lithuania, Saudi Arabia, Slovakia	10
(1, 1, 0)	Albania, United Arab Emirates, Austria, Cameroon, Costa Rica, Czech Republic, Algeria, Guyana, India, Israel, Kyrgyzstan, Maldives, Mauritania, Mauritius, New Zealand, Panama, Serbia, Sweden, Tanzania	19
(0, 1, 1)	Argentina, Denmark, Greece, Kenya, Cambodia, Kuwait, Laos, Myanmar, Paraguay, El-Salvador	10
(0, 1, 0)	Australia, Dominican Republic, Fiji, Croatia, Hungary, Nigeria, Nicaragua, Papua New Guinea, Poland, Russian Federation, Senegal	12
(1, 2, 0)	Burundi, Belgium, Bangladesh, Brazil, Congo, Egypt, Ghana, Gambia, Japan, Mali, Mozambique, Malawi, Malaysia, Peru, Philippines, Portugal, Tajikistan, Trinidad and Tobago, United States America	19
(0, 2, 0)	Benin, Bulgaria, Bahrain, Belize, Barbados, Canada, Switzerland, Chile, Côte d’Ivoire, Columbia, Germany, Spain, France, Honduras, Ireland, Iran, Liberia, Lesotho, Luxembourg, Latvia, Namibia, Niger, Norway, Pakistan, Qatar, Rwanda, Slovenia, Swaziland, Syrian Arab Republic, Togo, Thailand, Turkey, Uruguay, South Africa, Zambia	35
(0, 2, 1)	Bolivia, Central African Republic, Ecuador, Guatemala, Iraq, Italy, Mexico, Nepal, Tunisia, Ukraine, Zimbabwe	11
(0, 2, 2)	Botswana, China, Iceland, Republic of Moldova, Sierra Leone, Vietnam, Yemen	7
(1, 2, 1)	Democratic Republic of Congo, Indonesia, Romania, Uganda, Jordan	5
(3, 3, 0)	Cuba	1
(2, 1, 1)	Cyprus, Kazakhstan	2
(1, 0, 0)	United Kingdom	1
(2, 0, 0)	Republic of Korea	1
(2, 2, 2)	Sri Lanka	1
(2, 2, 1)	Latvia	1
(1, 1, 1)	Malta	1
(0, 1, 2)	Mongolia	1
(3, 1, 0)	Netherlands	1
(2, 0, 2)	Sudan, Singapore	1
(2, 2, 0)	Venezuela	1
Total Country		140

**Fig. 1:**
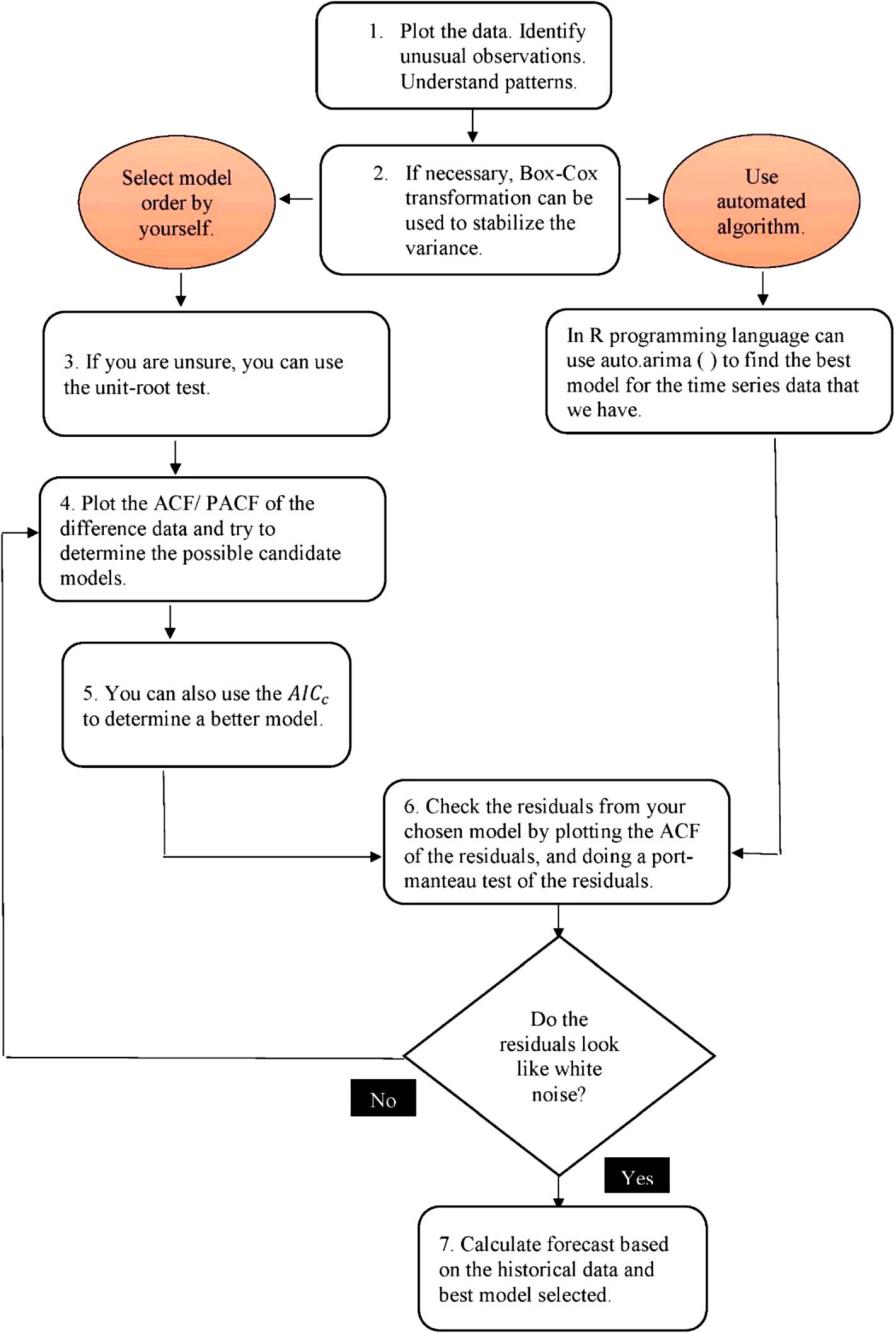
Steps performed in the forecasting using the ARIMA model in the R programming language

Box and Pierce [33] posited that ARIMA models are appropriate for long forecasting periods. We measure a future time series from 2016 to 2100 using the R programming language based on historical data measured from 1990 to 2015. Figure 1 summarizes the steps applied to forecast the health stock from 2016 to 2100. Using the trend component values of the time-series and demographic data projection by the UN, we can determine the global pattern of health stock in 140 countries.

## Results

### Heterogeneous growth of health stock (1990–2015)

**Fig. 2:**
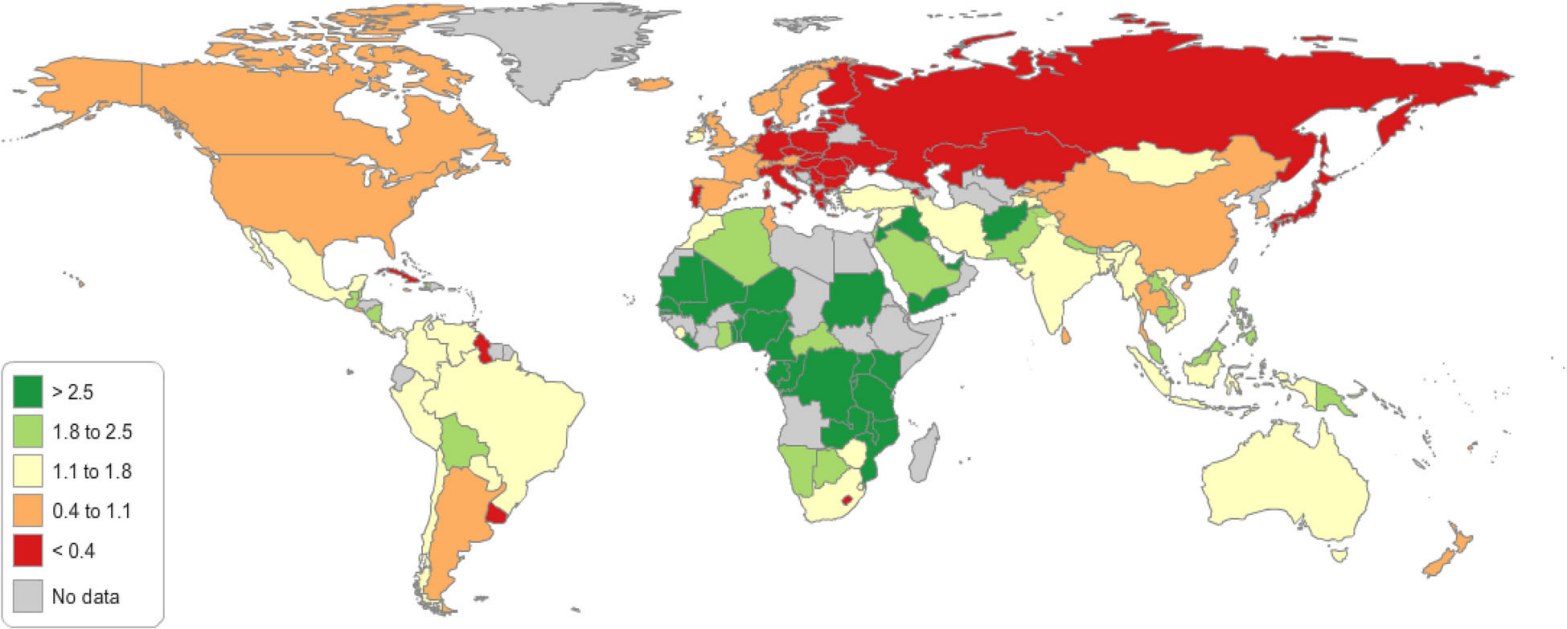
Health-stock growth (1990–2015)

First, we provide an overview of the global health-stock growth measured from 1990 to 2015. Over the study period from 1990 to 2015, most countries experienced gains in health stock. The health-stock growth from 1990 to 2015 is summarized in Fig. 2 and Table 3. The average health-stock growth rate across 140 countries is 1.55% per year during the sampled period.

**Table 3:** Ranking of average health-stock growth in 140 countries between 1990 and 2015

Ranking	Health-stock Growth	Average	Ranking	Health-stock Growth	Average
1	Qatar	6.89	71	Turkey	1.36
2	United Arab Emirates	4.86	72	Ireland	1.33
3	Bahrain	4.01	73	Morocco	1.32
4	Afghanistan	3.86	74	Myanmar	1.32
5	Jordan	3.65	75	Viet Nam	1.30
6	Niger	3.63	76	Peru	1.28
7	Uganda	3.59	77	Indonesia	1.27
8	Yemen	3.31	78	Colombia	1.26
9	Benin	3.29	79	Syrian Arab Republic	1.26
10	Congo	3.28	80	Brazil	1.23
11	Zambia	3.19	81	Chile	1.19
12	Liberia	3.10	82	Australia	1.18
13	United Republic of Tanzania	3.09	83	Zimbabwe	1.16
14	Democratic Republic of the Congo	3.09	84	South Africa	1.15
15	Gambia	3.03	85	Argentina	1.09
16	Mali	2.99	86	New Zealand	1.09
17	Rwanda	2.97	87	Iceland	1.05
18	Mozambique	2.91	88	Tunisia	1.02
19	Burundi	2.88	89	United States of America	1.01
20	Malawi	2.84	90	Sri Lanka	1.00
21	Togo	2.82	91	Jamaica	0.95
23	Iraq	2.78	92	Kyrgyzstan	0.92
23	Maldives	2.77	93	Norway	0.92
24	Kenya	2.76	94	Canada	0.86
25	Cameroon	2.73	95	Mauritius	0.83
26	Senegal	2.69	96	Spain	0.80
27	Nigeria	2.60	97	Switzerland	0.73
28	Singapore	2.58	98	Fiji	0.72
29	Mauritania	2.56	99	El Salvador	0.64
30	Sudan (former)	2.53	100	Thailand	0.61
31	Gabon	2.51	101	Sweden	0.61
32	Honduras	2.48	102	Republic of Korea	0.60
33	Papua New Guinea	2.46	103	Malta	0.54
34	Israel	2.46	104	United Kingdom	0.54
35	Kuwait	2.44	105	China	0.52
36	Côte d’Ivoire	2.41	106	Belgium	0.52
37	Belize	2.28	107	France	0.51
38	Central African Republic	2.28	108	Austria	0.48
39	Cambodia	2.25	109	Netherlands	0.47
40	Guatemala	2.15	110	Denmark	0.40
41	Botswana	2.14	111	Portugal	0.37
42	Saudi Arabia	2.14	112	Finland	0.36
43	Lao People’s Democratic Republic	2.13	113	Uruguay	0.35
44	Malaysia	2.12	114	Italy	0.28
45	Pakistan	2.11	115	Barbados	0.25
46	Ghana	2.08	116	Kazakhstan	0.20
47	Haiti	2.00	117	Czech Republic	0.15
48	Egypt	1.98	118	Greece	0.14
49	Bolivia	1.97	119	Lesotho	0.13
50	Namibia	1.95	120	Cuba	0.10
51	Philippines	1.93	121	Germany	0.07
52	Nepal	1.92	122	Slovakia	0.06
53	Cyprus	1.87	123	Poland	−0.01
54	Nicaragua	1.85	124	Slovenia	−0.10
55	Algeria	1.81	125	Japan	−0.11
56	Costa Rica	1.79	126	Hungary	−0.19
57	Paraguay	1.78	127	Guyana	−0.29
58	Tajikistan	1.78	128	Romania	−0.30
59	Swaziland	1.78	129	Russian Federation	−0.33
60	Luxembourg	1.72	130	Trinidad and Tobago	− 0.35
61	Ecuador	1.71	131	Albania	−0.37
62	Panama	1.65	132	Croatia	−0.44
63	Venezuela	1.61	133	Serbia	−0.47
64	Sierra Leone	1.49	134	Armenia	−0.66
65	Bangladesh	1.49	135	Ukraine	−0.73
66	India	1.49	136	Estonia	−0.89
67	Dominican Republic	1.48	137	Bulgaria	−0.95
68	Iran	1.43	138	Republic of Moldova	−0.97
69	Mongolia	1.39	139	Lithuania	−1.23
70	Mexico	1.38	140	Latvia	−1.28
Mean					1.55

**Fig. 3:**
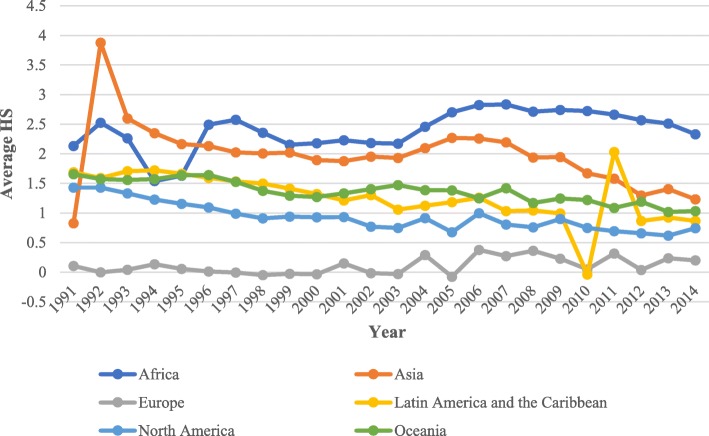
Average health-stock growth in six regions (1990–2015)

Figure 3 illustrates the cumulative average health-stock growth in the following six regions: Africa, Asia, Europe, Latin America and the Caribbean, North America, and Oceania. As indicated in Fig. 3, Africa clearly experienced the greatest cumulative growth, particularly since 1996, followed by Asia, Oceania, and Latin America and the Caribbean. North America and Europe have experienced the lowest cumulative growth. In 1992, Asia exhibited the highest average health stock growth, which drastically declined in 1993 and continued to decline until 2015. The decline in health stock that began in 1993 was due to the drastic decline in fertility rates. The fertility rates in Asia range from 4.5 to 2.1 children per woman, and several Asian countries have the lowest birth rates worldwide. For example, fertility rates in Korea have markedly declined over the last 50 years, and two parents in the current generation are replaced, on average, with only one child in the next generation. Thus, Korea has one of the lowest fertility rates among the Organization for Economic Co-operation and Development (OECD) countries [34]. By 1990, nine of 10 Asians were living in countries in which fertility had fallen by at least 25%, mainly due to the widespread use of contraception [35]. This decrease was also caused by delayed marriage and very low fertility [36]. During the prior few decades, several East Asian populations have joined Europe in the low-fertility league. Japan, Singapore, Taiwan, South Korea, and Hong Kong are among the ultra-low-fertility countries worldwide, and even China has reached fertility levels that are lower than those of European countries [37].

**Fig. 4:**
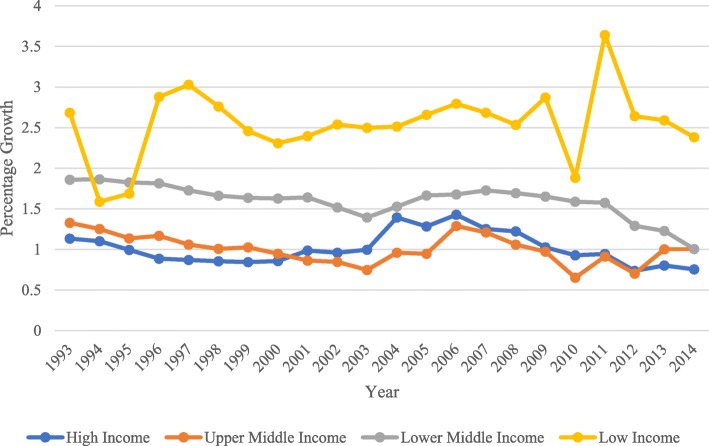
Average health-stock growth by country income (1993–2015)

Figure 4 illustrates that since 1995, LICs have exhibited the greatest health-stock growth, while high-income countries (HICs) have exhibited the lowest growth. Most LICs also exhibited the greatest growth during the study period due to improvements in health and population increases. The world has experienced enormous health improvements in the last century, particularly in its later half. However, despite the overall improvement, we also have to acknowledge that developing countries have benefited unequally from the abovementioned health gains, and many countries continue to have high mortality rates; in some parts of the world, the burden of ill health in the form of the infectious and parasitic disease is still prevalent [38]. Health and income inequality continue to exist among and within countries. For example, while HICs gain advantages in terms of life expectancy at birth, LICs and MICs struggle with disease and epidemics. Most lower-middle income countries (LMICs) struggle with chronic diseases, such as heart disease, HIV/AIDS, and cancer, and the risks increase with population ageing, urbanization, and the globalization of risk factors [39]. In LMICs, such as Sri Lanka, the availability of utility weights is of considerable importance because these countries require greater efficiency in health care resource allocation due to scarce resources and high disease burden [40].

As presented in Fig. 4, although LICs had the highest average growth of health stock and life expectancy age at birth, in terms of GDP, the LICs remain the lowest. The upper-middle-income countries (UMICs) exhibited the greatest increases in average GDP per capita and general life expectancy at birth globally. Life expectancy at birth has steadily increased globally over the prior few decades due to advancements in technology, medicine and international support [41]. Life expectancy today is higher than ever before with the modal lengths of life in low-mortality regions approaching 91 years for women and 86 years for men. At the global scale, LMICs are recording large declines in mortality at younger ages, while in HICs, the gains in life expectancy are due mainly to the decreasing trends in mortality rates among the elderly. With ever-increasing life expectancy globally, it is imperative for practitioners and policy-makers alike to build knowledge of how older peoples’ views of their own ageing, considering their health-related circumstances, affect their quality of life [42]. Furthermore, improvement in life expectancy most likely influences growth in health expenditure, and vice versa [43]. The decrease in fertility, particularly among HICs, also contributes to their low health stock.

### Global Health-stock forecasting (2016–2100)

**Fig. 5:**
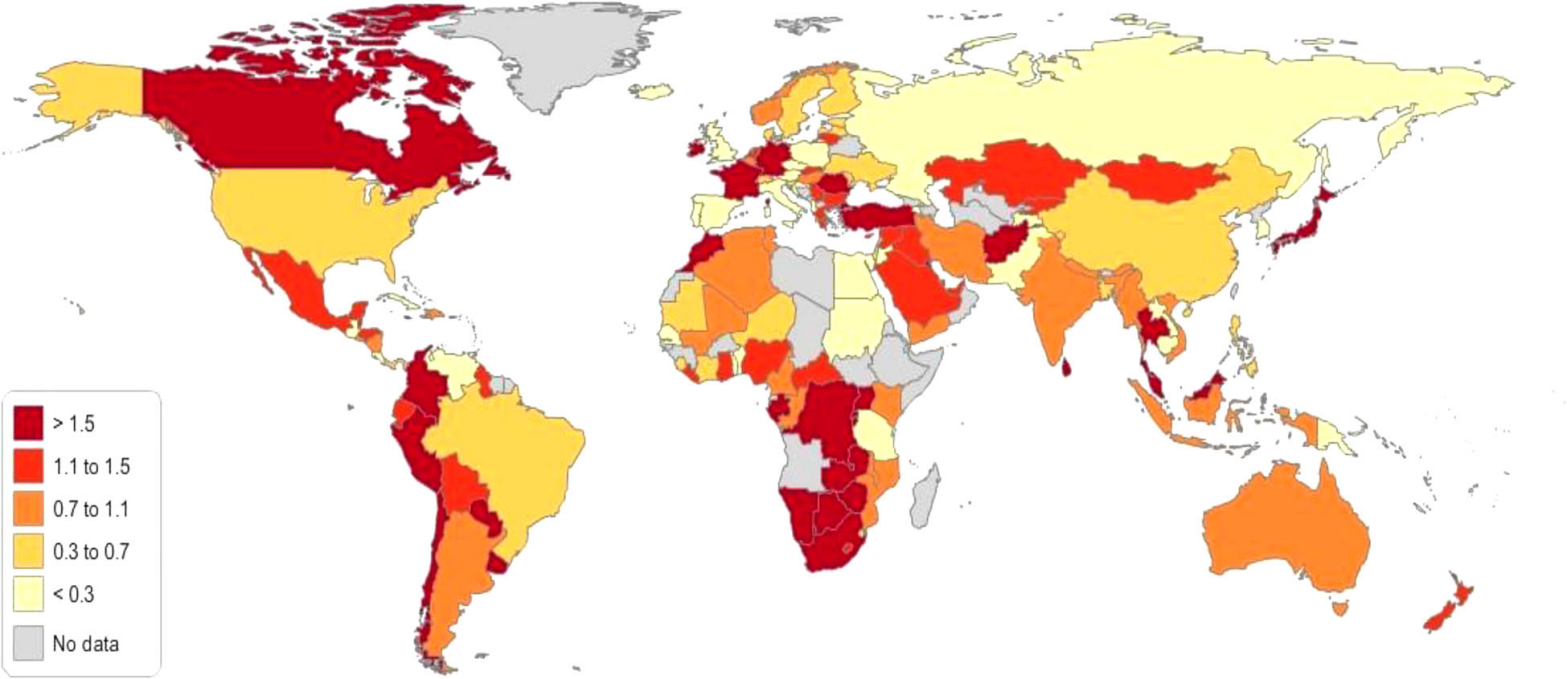
Average health-stock growth in 140 countries (1990–2100)

Using the previously measured health-stock values from 1990 to 2015, we projected the health-stock growth from 2016 to 2100 as in Fig. 5 and Table 3. Most countries in the study experienced gains in health stock during the period between 1990 and 2100. The average health-stock growth rate across 140 countries is 0.8% per year during the sampled period. As shown in Fig. 5, 121 countries, representing 86.4% of our sample, demonstrated positive health-stock growth. Table 4 presents the magnitude of health-stock growth in 140 countries from 1990 to 2100 in descending order.

**Table 4:** Ranking and summary of the estimated forecast health-stock growth (1990–2100)

Ranking	Country	Health-stock Growth (%)	Ranking	Country	Health –stock Growth (%)	Ranking	Country	Health-stock Growth (%)
1	Peru	2.65	48	Mexico	1.23	95	Switzerland	0.59
2	Turkey	2.12	49	Belize	1.21	96	Slovenia	0.58
3	Malaysia	2.09	50	Bolivia (Plurinational State of)	1.21	97	Estonia	0.56
4	Uganda	2.08	51	Mongolia	1.20	98	Republic of Moldova	0.52
5	Sri Lanka	2.04	52	United Arab Emirates	1.20	99	Swaziland	0.50
6	Uruguay	1.96	53	Syrian Arab Republic	1.20	100	Ukraine	0.49
7	Zambia	1.93	54	New Zealand	1.19	101	Niger	0.43
8	Burundi	1.88	55	Guyana	1.18	102	Fiji	0.43
9	Ireland	1.88	56	Lesotho	1.16	103	Mauritania	0.39
10	Luxembourg	1.87	57	Qatar	1.16	104	Philippines	0.39
11	Afghanistan	1.87	58	Cyprus	1.15	105	Denmark	0.33
12	Botswana	1.86	59	Slovakia	1.13	106	Finland	0.33
13	Bahrain	1.86	60	Singapore	1.10	107	Panama	0.31
14	South Africa	1.81	61	Algeria	1.07	108	Latvia	0.31
15	Chile	1.80	62	Nicaragua	1.06	109	Côte d’Ivoire	0.31
16	Germany	1.77	63	India	1.02	110	Egypt	0.26
17	Jamaica	1.76	64	Viet Nam	1.00	111	Togo	0.26
18	Rwanda	1.76	65	Hungary	0.99	112	Iceland	0.24
19	Canada	1.75	66	Congo	0.98	113	Venezuela (Bolivarian Republic of)	0.24
20	Namibia	1.75	67	Dominican Republic	0.98	114	Austria	0.20
21	Thailand	1.71	68	Mozambique	0.98	115	Czech Republic	0.14
22	D.R. of the Congo	1.70	69	Malawi	0.96	116	Benin 0.13	
23	Romania	1.66	70	Tunisia	0.96	117	Jordan	0.12
24	Kuwait	1.64	71	Iran (Islamic Republic of)	0.95	118	Cuba	0.02
25	Zimbabwe	1.62	72	Norway	0.94	119	United Kingdom	0.01
26	Gabon	1.62	73	Maldives	0.94	120	Republic of Korea	0.01
27	Malta	1.57	74	Myanmar	0.90	121	Sudan (Former)	0.01
28	Paraguay	1.57	75	Cameroon	0.88	122	Gambia	−0.02
29	Japan	1.55	76	Belgium	0.87	123	Albania	−0.04
30	Morocco	1.53	77	Australia	0.83	124	Portugal	−0.08
31	France	1.53	78	Nepal	0.81	125	Senegal	−0.12
32	Colombia	1.50	79	Mali	0.79	126	Costa Rica	−0.14
33	Kazakhstan	1.50	80	Argentina	0.78	127	Pakistan	−0.15
34	Lithuania	1.48	81	Mauritius	0.78	128	Croatia	−0.21
35	Ecuador	1.47	82	Indonesia	0.77	129	Guatemala	−0.31
36	Ghana	1.46	83	Haiti	0.77	130	Poland	−0.35
37	Saudi Arabia	1.45	84	Yemen	0.75	131	Italy	−0.36
38	Serbia	1.44	85	El Salvador	0.74	132	Russian Federation	−0.60
39	Liberia	1.39	86	Trinidad and Tobago	0.73	133	Armenia	−0.72
40	Netherlands	1.38	87	Kenya	0.70	134	Spain	−0.83
41	Iraq	1.36	88	China	0.69	135	Barbados	−0.94
42	Bulgaria	1.36	89	Brazil	0.68	136	U.R. of Tanzania: Mainland	−1.19
43	Honduras	1.34	90	Israel	0.68	137	Cambodia	−1.25
44	Greece	1.29	91	United States	0.67	138	Tajikistan	−1.41
45	Kyrgyzstan	1.27	92	Sierra Leone	0.62	139	Lao People’s DR	−2.36
46	Nigeria	1.26	93	Bangladesh	0.61	140	Papua New Guinea	−2.69
47	Central African Republic	1.25	94	Sweden	0.60	Average		0.8

**Fig. 6:**
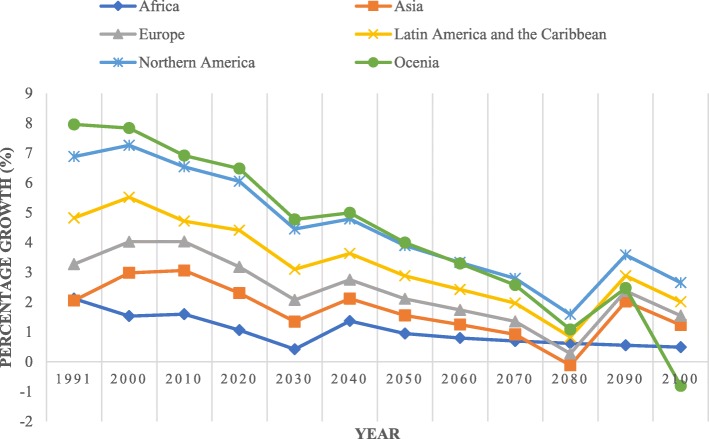
Historical and forecasted health-stock growth by region (1990–2100)

Figure 6 depicts the historical (1990–2015) and forecasted health stock growths from 1990 to 2100 by region. As illustrated in Fig. 6, since 2000, the Oceania region has had highest health stock growth followed by North America, Latin America, and the Caribbean. Africa exhibited the lowest health-stock growth between 1990 and 2100. We projected that the health-stock growth in all regions will decrease, particularly in Africa in 2030 and Asia in 2080. The factors causing the declines in health stock include fertility declines and population ageing. These effects arise because countries in Asia, Latin America and the Caribbean experienced an accelerated fertility decline of more than one birth per five-year time period [44]. Several Asian countries, including Japan, Singapore, Taiwan, South Korea, and Hong Kong, are the ultra-low-fertility countries worldwide, and even China has reached a lower level of fertility than many European countries [37]. Asia, particularly the Pacific OECD, is likely to shrink in population size and experience extreme population ageing. The proportion of the population aged 60 years and older in these countries (with Japan having the greatest weight) is expected to reach 50% of the total population. The China region will experience more rapid ageing, and the proportion of the population aged 60 and older is expected to increase by a factor of four from 10% in 2000 to 39% in 2100 [45].

**Fig. 7:**
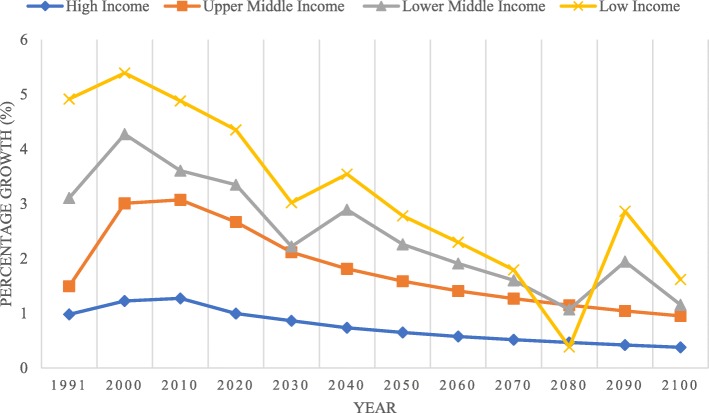
Health-stock growth by income region (1990–2100)

We also compare the population and total fertility growth in each country based on the income group. As shown in Fig. 7, since 2000, LICs have the highest health-stock growth, followed by LMICs. Although LICs exhibit the highest average population and fertility growth, they show the lowest health-stock growth during the study period. Most income groups show declining fertility. HICs exhibit the lowest health-stock growth from 1990 to 2100. We projected that health-stock growth on all the continents will decrease, particularly in LICs, which will drastically decrease in 2030 and 2080. The estimated health-stock decline in 2030 and 2080 in LICs is approximately 0.8% and 0.7%, respectively. UMICs and HICs will exhibit a gradual decrease in their health-stock growth by an average of 1.04 and 0.76% per year, respectively.

**Fig. 8:**
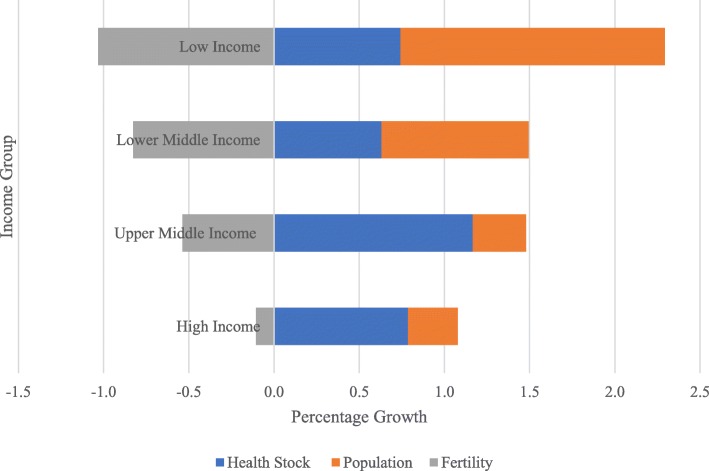
Health-stock, population and fertility growth by income region (1990–2100)

Using the demographic data projected by the UN, we analysed how demographic factors contribute to forecasted values of health stock from 1990 to 2100. Figure 8 depicts the average health stock, fertility and population growth from 1990 to 2100 based on income groups. In this study, during the sampling period between 1990 and 2015, LICs had the highest mean growth in health scores, while HICs exhibited the lowest mean growth. However, in the long-term from 1990 to 2100, UMICs, on average, have the highest projected health-stock growth, followed by HICs and LICs because most UMICs, particularly in the Middle East and North Africa (MENA), are expected to reach their peak youth population of approximately 94 million in 2030. The number of youth aged 15 to 24 years in Iraq is expected to double over the next 30 years [46]. The ‘youth bulge’ experienced in the MENA region poses opportunities as well as challenges for development. For example, with two-thirds of its population between 15 and 29, the MENA region has one of the largest youth groupings in the world. High fertility rates mean that many more will join this cohort over the next two decades [47]. Although this shift should imply a huge economic opportunity for the region and turn the youth bulge into a demographic dividend, the youth in this region might be seen as a burden on the economy, which has to provide more health care services in addition to decent job opportunities [48].

Other than the fertility rate, population ageing, and working-age and youth populations, immigration may be a factor contributing to health-stock growth. For instance, compared to LICs and LMICs, although the population and fertility growth in UMICs and HICs are the smallest in terms of health stock, greater progress is observed.

## Discussion

In this study, we presented a quantitative evaluation of health capital based on measurements of health stock from 1990 to 2015, and we presented time-series forecasting of the global health stock in 140 countries from 2016 to 2100.

We identified significant differences in the health stock values among all 140 countries based on population, fertility, mortality, working-age population, life expectancy, the stability or instability of the country, and the balance of immigration and emigration. It may be clear if we observe country-level results from 1990 to 2015, e.g., Qatar and the UAE had the highest average health stock growth due to incoming migrants. In addition to it, population ageing and declines in fertility and population, which challenge the well-being of societies and countries, particularly HICs, can be addressed. In this study, during the sampling period between 1990 and 2015, LICs exhibited the highest mean growth in health scores, while HICs exhibited the lowest mean growth due to improvements in health and population increases, particularly in LICs. However, in the long-term, from 1990 to 2100, the UMICs, on average, are projected to exhibit the highest average health stock growth because most UMICs, particularly in the MENA, are expected to reach their youth population peaks in 2030.

We also applied a time-series model to forecast the global health stock from 2016 to 2100 using historical health stock data measured from 1990 to 2015. The trends in the forecasted values from 1990 to 2100 revealed that most countries have projected increases in health stock, particularly UMICs. Compared to LICs and LMICs, the population and fertility growths in UMICs and HICs are the smallest; however, in terms of health stock, the latter countries exhibit more progress. In addition to the fertility rate, population ageing and the working-age and youth populations, immigration may be a contributing factor to health stock growth. Most countries in the study experienced health stock gains during the period from 1990 to 2100. The average health-stock growth rate across the 140 countries was 0.8% annually during the sampled period.

## Conclusions

This study clarified that our measurement of national health stock under the capital approach have an ability of revealing heterogeneous stock allocations in the world. The indicator also demonstrated that 121 in 140 countries are expected to be sustainable for the human health, since the increment of health capital stock is a positive signal for sustainability. These findings support the useful of the health stock indicator as a component of sustainability indexes.

## Abbreviations

ACF: Auto-correlation function
AR: Autoregressive
ARIMA: Autoregressive integrated moving average
HICs: High-income countries
IWR 2012: Inclusive Wealth Report 2012
LICs: Low-income countries
LMICs: Lower-middle income countries
MA: Moving average
MENA: Middle East and North Africa
OECD: Organization for Economic Co-operation and Development
PACF: Partial auto-correlation function
SDGs: Sustainability Development Goals
UAE: United Arab Emirates
UMICs: upper-middle-income countries
UN: United Nations
VSL: Value of statistical life
WHO: World Health Organization
